# Pancreatic Mass in a Patient with a History of Resected Renal Cell Carcinoma and Resected Adenocarcinoma of the Ampulla of Vater: A Case Report

**DOI:** 10.1089/pancan.2018.0001

**Published:** 2018-08-01

**Authors:** Sarah M. Kling, Sami Tannouri, Wei Jiang, Charles J. Yeo

**Affiliations:** Department of Surgery, Sidney Kimmel Medical College at Thomas Jefferson University Hospital, Philadelphia, Pennsylvania.

**Keywords:** metastases, pancreatic adenocarcinoma, renal cell carcinoma

## Abstract

**Background:** Metastases of renal cell carcinoma (RCC) to the pancreas are rare, whereas recurrence of pancreatic ductal adenocarcinoma (PDA) or a primary periampullary cancer is far more common. The time elapsed between a primary tumor and a new mass can aid in differentiation between the two.

**Presentation:** A 70-year-old man with a history of RCC status after left nephrectomy and ampullary adenocarcinoma status after pancreaticoduodenectomy presents with an incidentally found mass in his remnant pancreas. Resection of the mass via completion pancreatectomy yielded pathology consistent with metastatic RCC.

**Conclusions:** Metastases of RCC to the pancreas often present many years after a primary resection. Conversely, recurrent PDA often presents within 5 years of resection. Resection of RCC metastases yields better survival than resection of recurrent PDA, which is controversial. We recommend resection of suspected isolated pancreatic RCC metastases due to known favorable outcomes.

## Introduction

Renal cell carcinoma (RCC) makes up 3–6% of all cancers in the United States.^1^ RCC is the most common subtype of adult kidney cancer and accounts for 3% of all newly diagnosed malignancies.^1,2^ RCC carries a 5-year survival rate of 95% when localized to the kidney.^1^ Surgical resection of the primary RCC is both standard treatment and curative in the absence of metastatic disease.^3^ Metastases occur in 30–50% of cases, even after resection of localized primary disease, and decrease 5-year survival to 0–20%.^1,3^ On computed tomography (CT) imaging, RCC metastases are typically hypervascular with contrast enhancement, multicentric, and without peripancreatic lymphadenopathy.^2^

Pancreatic ductal adenocarcinoma (PDA) has an incidence of 1.1–1.2% in the United States.^4^ PDA is one of the most fatal cancers and is the third leading cause of cancer-related death.^4,5^ Surgical resection is standard treatment and is the only chance for cure; however, 5-year survival rates after resection remain low (20%).^5^ PDA also harbors a high incidence of local recurrence (72–86%) following resection.^4,5^ On CT imaging, PDA is usually hypointense in the contrast phase.^2^

## Case

**FIG. 1.**
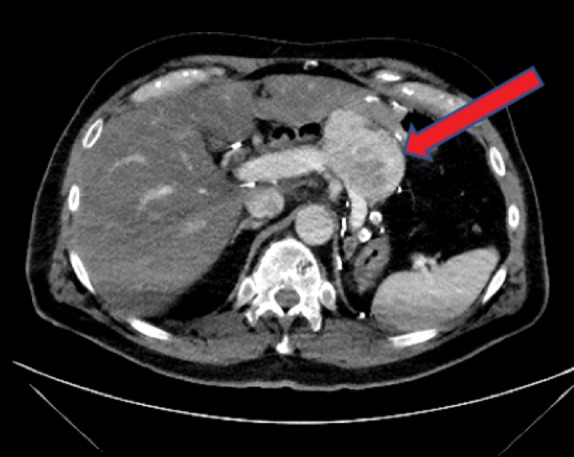
Abdominal CT scan revealing a 7 cm hypervascular mass (arrow) involving the splenic vein. Typically, RCC metastases are hypervascular with contrast enhancement, multicentric, and without peripancreatic lymphadenopathy.^2^ CT, computed tomography; RCC, renal cell carcinoma.

A 70-year-old man presented in 2017 after discovery of a hypervascular, expansile, 7 cm pancreatic body mass involving the splenic vein on routine surveillance CT scan (Fig. 1). He endorsed 1 year of diarrhea and steatorrhea that he had not sought medical care for but denied weight loss, appetite changes, fatigue, pain, or jaundice. He had a history of RCC and underwent a left nephrectomy and adrenalectomy in 2002. Additionally, he developed adenocarcinoma of the ampulla of Vater and was treated with pancreaticoduodenectomy in 2003.

He developed pulmonary metastases from the RCC and was treated with right lung segmentectomy in 2006 and three bilateral CyberKnife treatments in 2010, 2011, and 2013. He had surveillance CT scans roughly every 6 months from the discovery of the 2010 lung metastasis until 2015 and then yearly thereafter. He never received any chemotherapy for either the RCC or PDA. He is a former smoker.

Unfortunately, records of his prior malignancies, treatments, and surgeries were limited. His metastatic workup consisted of a CT scan of the head, chest, abdomen, and pelvis, which revealed no other sites of tumor except the 7 cm mass in the body of the pancreas, and testing his carcinoembryonic antigen (CEA) tumor marker level, which resulted as 0.9 ng/mL. The two main differential diagnoses were metastatic RCC versus recurrent PDA. Imaging characteristics, timeline of the mass development, and normal CEA level were the investigations used to determine that metastatic RCC was most likely and supported the decision to resect the mass.

The patient underwent completion pancreatectomy and splenectomy to resect his newly discovered pancreatic lesion. Surgical exploration and dissection revealed that the previous pancreaticoduodenectomy had been completed entirely antecolic. The gastrojejunostomy was able to be left intact throughout the duration of the procedure, whereas the hepaticojejunostomy was reconstructed due to its initial position being too far anterior in relation to the current surgical bed. Due to vascular invasion, a part of the portal superior mesenteric–splenic vein junction was resected with primary venovenostomy. His postoperative course was uncomplicated, and he was discharged on postoperative day 6.

Due to his apancreatic status, he received diabetic teaching, was commenced on parenteral insulin, and referred to an endocrinologist for follow-up. He was also counseled on pancreatic enzyme supplementation and scheduled to have repeat CT scans at 4 months postoperatively, and then, every 3 months thereafter provided no active disease progression. Due to his splenectomy and asplenic status, he received appropriate vaccinations for encapsulated organisms: Pneumococcus, Meningococcus, and *Haemophilus Influenzae*.

**FIG. 2.**
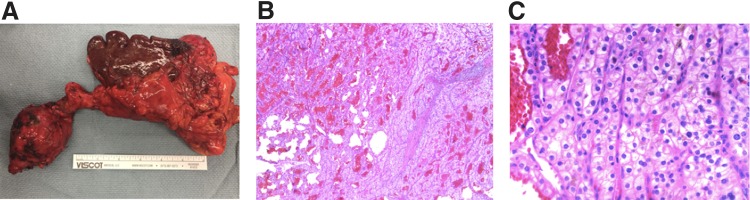
**(A)** Gross specimen including the pancreatic mass (left) and spleen (right). **(B)** Histology of pancreatic mass (H&E, 20× ). At lower power, the tumor cells are arranged in alveolar and solid growth pattern, with rich network of small thin-walled blood vessels. **(C)** Histology of pancreatic mass (H&E, 200× ). At higher power, the lesional cells have clear cytoplasm with distinct cell borders, with large and round nuclei. H&E, hematoxylin and eosin.

Grossly, a 6.5 × 6.2 × 4.5 cm well-circumscribed mass is located within the pancreatic parenchyma (Fig. 2A). On cut surface, it is glistening, red-purple, and slightly lobulated, with foci of hemorrhage. Histologically, the tumor has alveolar and solid growth pattern, with rich network of small thin-walled blood vessels (Fig. 2B). At higher power, the lesional cells have clear cytoplasm with distinct cell borders, with large and round nuclei (Fig. 2C). The morphological features in conjunction with the history are consistent with a metastatic clear cell RCC. The margins are negative, and 1/6 lymph nodes is positive for metastatic carcinoma.

The patient followed up with his medical oncologist who advised against adjuvant chemotherapy or radiation therapy, opting for imaging surveillance for now despite the one positive lymph node.

## Discussion

### Isolated pancreatic metastases from RCC

RCC most commonly metastasizes to the lung, liver, brain, adrenals, bone, and rarely to the pancreas; however, it is the most common primary tumor to metastasize to the pancreas.^1,2^ Patients with metastatic RCC are found to have metastases to the pancreas, with an incidence of 2–11%.^1^ Isolated RCC metastases to the pancreas typically occur many years after resection of the primary tumor, with a mean of 11.2 years and median of 6–12 years,^3^ and a mean of 10 ± 6.5 years in another study.^6^

After resection of pancreatic RCC metastases, the median survival is 5.5 years, with a 5-year survival rate of 51.8%.^3^ Another study noted 3- and 5-year survival rates of 21% and 0%, respectively, for patients with nonresected metastases versus 78% and 72%, respectively, for resected patients.^6^ The interval between nephrectomy to pancreatic metastasectomy is not predictive of overall survival.^3^ Therefore, surgical resection of isolated pancreatic metastases of RCC is effective and should be considered when identified.

Surgery is the mainstay of treatment for RCC and its metastases.^7^ The role of adjuvant chemotherapy for metastatic RCC is controversial due to RCC classically being refractory to chemotherapy.^8^ It is believed that RCC chemoresistance stems from a variety of gene mutations and changes in gene expression.^8^ In addition to chemotherapy, various targeted therapies and immunotherapies have been developed and trialed in the treatment of RCC, which have also proven to be controversial in their abilities to treat RCC.^7,8^

It is noted that 20–25% of patients with metastatic RCC are refractory to initial treatment with these therapies.^7^ Of those who do respond initially, the majority will eventually experience disease progression, which is thought to be due to the development of resistance.^7^ Metastatic RCC remains as an incurable disease despite the use of chemotherapy, targeted therapy, and immunotherapy, and the use of these therapies is not necessarily indicated even in patients with surgically controlled disease.^7,8^

### Primary pancreatic cancer recurrence

Within 5 years of resection of PDA, 80% of patients will recur, with >60% recurring within 2 years.^4^ In one study, recurrent PDA had a median survival time of 7 months from detection of recurrence until death.^5^ Considerations for re-resection include isolated nonvascular recurrence within the remnant pancreas, and the patient must be able to tolerate being apancreatic.^5^ The median survival time after re-resection of recurrent PDA in highly selected patients ranges from 25 to 27.5 months, versus 9–10.8 months for nonresected patients^4,5^ and 15.9–17.5 months in patients who received only chemoradiation for recurrence.^5^

## Conclusion

Metastases of RCC to the pancreas are rare, whereas recurrence of PDA is much more common. Resection of RCC metastases yields favorable survival results, whereas resection of recurrent PDA is controversial. Timing of presentation can help differentiate new pancreatic masses in patients with histories of RCC and PDA. This patient's presentation with a history of RCC, long interval to recurrence, and radiological findings supported the belief that his new pancreatic mass was metastatic RCC and not recurrent PDA. This led to the decision to resect the mass with a completion pancreatectomy.

## Abbreviations Used

CEA: carcinoembryonic antigen
CT: computed tomography
H&E: hematoxylin and eosin
PDA: pancreatic ductal adenocarcinoma
RCC: renal cell carcinoma
